# [^99m^Tc]-labelled anti-Programmed Death-Ligand 1 single-domain antibody SPECT/CT: a novel imaging biomarker for myocardial PD-L1 expression

**DOI:** 10.1186/s13550-023-00990-7

**Published:** 2023-05-17

**Authors:** Muhummad Sohaib Nazir, Daniel Johnathan Hughes, Gitasha Chand, Kathryn Adamson, Jessica Johnson, Damion Bailey, Victoria Gibson, Hong Hoi Ting, Alexander R. Lyon, Gary J. R. Cook, PECan study group, Scott Edmonds, Alexandros Georgiou, Eleni Karapanagiotou, Debra Josephs, Emma McLean, James Spicer, Vicky Goh

**Affiliations:** 1 Department of Cardiovascular Imaging, School of Biomedical Engineering and Imaging Sciences, King’s College London, London, SE1 7EH UK; 2 Royal Brompton Hospital, Guy’s and St Thomas’ NHS Foundation Trust, London, UK; 3 Department of Cancer Imaging, School of Biomedical Engineering and Imaging Sciences, King’s College London, London, UK; 4 King’s College London & Guy’s and St Thomas’ PET Centre, London, UK; 5 King’s Health Partners Comprehensive Cancer Centre, Guy’s and St Thomas’ NHS Foundation Trust, London, UK; 6 Nanomab Technology (UK) Limited, London, UK; 7 Department of Nuclear Medicine, Guy’s and St Thomas’ NHS Foundation Trust, London, UK; 8 National Heart & Lung Institute, Imperial College London, London, UK

**Keywords:** SPECT-CT, Immune checkpoint inhibitor, PD-L1 expression

## Abstract

**Background:**

Myocardial programmed death-ligand 1 (PD-L1) expression is implicated in immune checkpoint inhibitor (ICI)-associated myocarditis. Measurement of myocardial PD-L1 expression may have potential use as a mechanistic and predictive biomarker. The aim of this study was to determine non-invasive assessment of myocardial PD-L1 expression using [^99m^Tc]-labelled anti-PD-L1 single-domain antibody (NM-01) SPECT/CT.

**Methods:**

Thoracic [^99m^Tc]NM-01 SPECT/CT was performed in lung cancer patients (*n* = 10) at baseline and 9-weeks following anti-programmed cell death protein 1 (PD-1) therapy. Baseline and 9-week left ventricular and right ventricular to blood pool ratios (LV_max_:BP) and (RV_max_:BP) were measured. LV_max_ was compared to background skeletal muscle (muscle_max_). Intra-rater reliability was determined by intraclass correlation coefficient (ICC) and Bland–Altman analysis.

**Results:**

Mean LV_max_:BP values were 2.76 ± 0.67 at baseline vs 2.55 ± 0.77 at 9 weeks (*p* = 0.42). Mean RV_max_:BP was 1.82 ± 0.32 at baseline vs 1.76 ± 0.45 at 9 weeks (*p* = 0.67). Myocardial PD-L1 expression was at least threefold greater than skeletal muscle at baseline for the LV (LV_max_ to muscle_max_ 3.71 ± 0.77 vs 0.98 ± 0.20 (*p* < 0.001)) and at least twofold for the RV (LV_max_ to muscle_max_ 2.49 ± 0.63 vs 0.98 ± 0.20 (*p* < 0.001)). There was excellent intra-rater reliability for LV_max_:BP with ICC 0.99 (95% confidence interval 0.94–0.99, *p* < 0.001), mean bias -0.05 ± 0.14 (95% limits of agreement -0.32 to 0.21). There were no major adverse cardiovascular events or myocarditis during follow-up.

**Conclusion:**

This study is the first to report PD-L1 expression of the heart that can be quantified non-invasively without invasive myocardial biopsy, with high reliability and specificity. This technique can be applied to investigate myocardial PD-L1 expression in ICI-associated myocarditis and cardiomyopathies.

*Clinical trial registration* PD-L1 Expression in Cancer (PECan) study (NCT04436406). https://clinicaltrials.gov/ct2/show/NCT04436406 June 18th, 2020.

## Background

Immune checkpoint inhibitor (ICI) therapy has revolutionized the treatment of cancers such as melanoma, renal cell carcinoma and non-small cell lung cancer (NSCLC) [1–3]. Cytotoxic T cell activity is attenuated by suppressive pathways, including programmed cell death protein 1 (PD-1). Various cancer cells overexpress programmed cell death-ligand 1, which on interaction with PD-1 leads to immune cell downregulation and as such, evasion of the host cytotoxic immune response [4]. ICIs work by blocking these inhibitory pathways, activating the T cell-mediated anti-cancer immune response.

Despite the impressive and durable responses seen in many cancers, ICIs are associated with immune-related adverse effects, such as colitis, pneumonitis and hepatitis [5]. Less frequently, ICIs can cause myocarditis with fatality rates of up to 50% reported [5]. According to one registry, the prevalence of ICI-associated myocarditis has been reported to be 1.14%, with a median time of onset of 34 days after initiation of therapy, and more commonly occurs in patients receiving combination ICI therapy [6].

The precise mechanism of ICI-associated myocarditis is not well understood, but PD-L1 expression has been implicated [7]. However, there are no validated methods to assess PD-L1 expression in the human heart. The feasibility and specificity of [^99m^Tc]-labelled anti-PD-L1 single-domain antibody (NM-01) single-photon emission computed tomography/computed tomography (SPECT/CT) for PD-L1 expression have been demonstrated in preclinical and clinical studies, and the monospecificity and high affinity of NM-01 to human PD-L1 with no off-target binding have been confirmed [8, 9]. Additionally, early data from the ongoing PD-L1 Expression in Cancer (PECan) clinical trial (NCT04436406) demonstrated an association between high baseline PD-L1 expression, determined by [^99m^Tc]NM-01 SPECT/CT, and 9-week ICI-treatment response in advanced NSCLC [10]. In this current study, we report the cardiac substudy findings of the ongoing PECan study. We sought to determine the feasibility of non-invasive assessment of PD-L1 expression in the myocardium using this novel tracer, [^99m^Tc]NM-01 SPECT/CT, in patients undergoing anti-PD-1 therapy for NSCLC.

## Methods

In this single-centre prospective observational study, performed at Guy’s and St Thomas’ NHS Foundation Trust, patients aged ≥ 18 years with advanced NSCLC scheduled to commence anti-PD-1/PD-L1 therapy ± chemotherapy were eligible. Exclusion criteria included patients with a prognosis < 3 months and/or Eastern Cooperative Oncology Performance (ECOG) status (≥ 2), previous ICI therapy, systemic anti-cancer therapy within preceding 14 days and pregnant and lactating women. Participants provided written informed consent, and the study was conducted with approval from the United Kingdom Research Ethics Committee and Health Research Authority (reference 256684).

Tracer preparation: ^99m^Tc-triaquatricarbonyltechnetium(I) [^99m^Tc(OH_2_)_3_(CO)_3_]^+^ intermediate (pH 7.0–8.0) is added to 200 μg of NM-01 in 100μL of phosphate-buffered saline (pH 7.4). The mixture is incubated at 37 °C for 1 h to give ^99m^Tc-NM-01. The contents are diluted in physiological saline to 2.0 mL and passed through two 0.22-μm filters before quality control testing.

A final drug product with a radiochemical purity of more than 90%, pH between 6.5–7.5, endotoxin levels < 20 EU/vial with a colourless, clear appearance is deemed acceptable for use and released for patient injection within its shelf life of 6 h.

Patients were administered 370–740 MBq intravenous [^99m^Tc]NM-01 with SPECT/CT performed 2 h post-injection, at baseline and at 9 weeks following initiation of anti-PD-1/PD-L1 therapy. Imaging was performed 2 h post-administration following our findings from our Phase 1 study indicating this to be the optimal time period for acquisition [9]. Single field-of-view thoracic SPECT/CT scans were performed on a Siemens Intevo SPECT/CT scanner with low-energy high-resolution collimators 256 × 256 matrix, 128 projections (64 views) over 180° rotation, 20 s per projection. A low-dose CT scan (110 kV, 25 mA, CTDI average 5.55 mGy, DLP average 246 mGy.cm) was acquired for anatomical detail and attenuation correction using BroadQuant (Siemens, Erlangen, Germany).

**Fig. 1:**
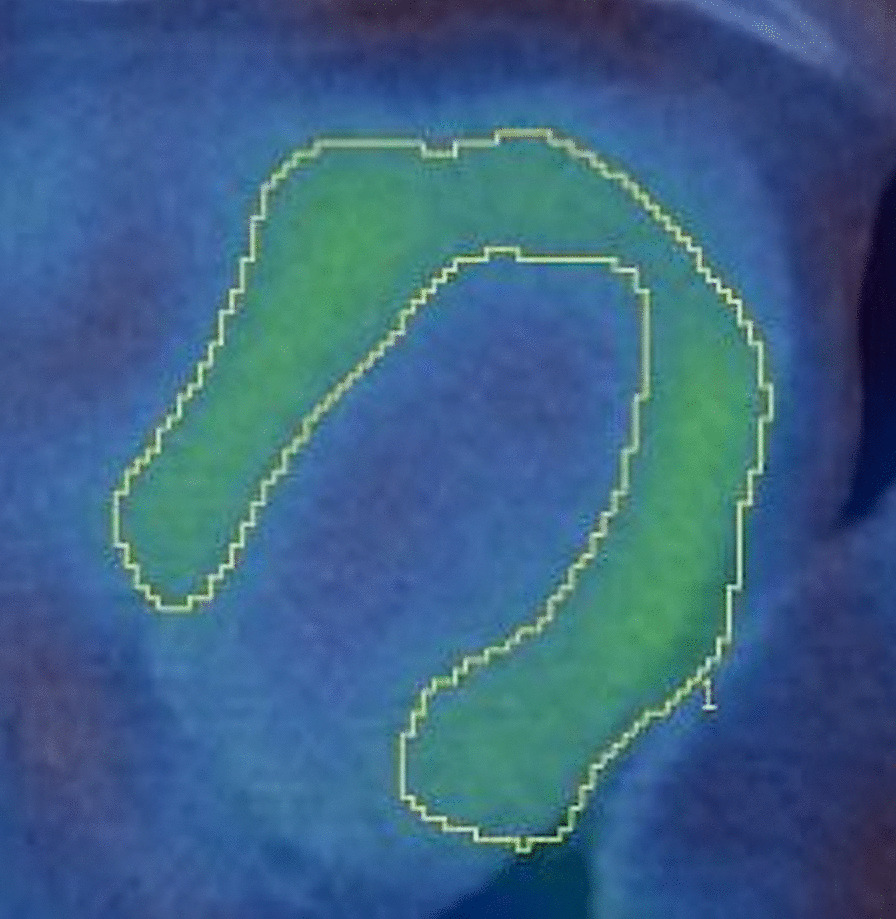
Sample image of region of interest of the myocardium for quantitative assessment of myocardial PD-L1 expression

Images were analysed by an experienced nuclear imaging specialist with 32 years of experience using Hermes GOLD™ software (Hermes Medical Solutions; Stockholm, Sweden). Baseline and 9-week left ventricular (LV), right ventricular (RV) and blood pool (BP) mean and maximum region-of-interest (ROI) values were measured by drawing regions of interest of the LV and RV myocardium and mediastinal blood pool (aortic arch) as shown in Fig. 1. LV_max_:BP and RV_max_:BP ratios were calculated for each SPECT/CT scan. Measurements were repeated by the same rater after a three-month period to assess for intra-rater reliability. A quantitative measure of heterogeneity, heterogeneity index (HI), was calculated as a ratio ROI_max_: ROI_mean_ for both the left ventricle (LV_max_) and right ventricle (RV_max_).

PD-L1 is constitutively expressed in a range of tissues, which include hematopoietic, vascular endothelial and pancreatic islet cells and in sites of immune privilege such as the placenta, testes and eyes [11]. Skeletal muscle has not been reported to have high levels of PD-L1 expression [12]. Thus, the maximum ROI of skeletal muscle (muscle_max_) was used as a reference region to compare with LV_max_ and RV_max_, as a marker of target specificity of the tracer. For consistency, the muscle_max_ ROI was taken from the left paravertebral muscle at the axial level of the mid left ventricle.

Patients underwent standard clinical follow-up during therapy and were observed for major adverse cardiovascular events (MACE) (defined by acute myocardial infarction, acute heart failure or cardiovascular death), or acute myocarditis (such as chest pain, fever, raised serum troponin and imaging findings) as defined in international guidelines [13].

Statistical analyses were performed using SPSS Statistics 27 (IBM, Armonk, NY, USA). Normality was assessed using the Shapiro–Wilk’s test. Results are expressed as mean ± standard deviation unless otherwise specified. Parametric Student t-tests were used to compare paired means for continuous normally distributed data and nonparametric Wilcoxon signed-rank test for continuous non-normally distributed data. Intra-rater reliability was assessed using intraclass correlation coefficients (ICC) using a two-way mixed absolute agreement model and Bland-and-Altman analyses [14, 15]. Two-tailed values of *p* < 0.05 were considered statistically significant.

## Results

Ten patients (median 64 years; 6 males) were included. The demographic and clinical characteristics of the patients and baseline values are shown in Table 1, and the baseline SUVs are shown in Table 2**.** All 10 patients completed the baseline SPECT/CT. One patient died as a result of ICI-related pneumonitis, a recognized side effect of anti-PD-1 therapy, and therefore did not have a second SPECT/CT.

**Table 1:** Demographic and clinical characteristics of patients

Patient	Age	Gender	BMI (kg/m^2^)	Clinical staging	Tumour PD-L1 expression (%)	ECOG performance status	Smoking status	Cardiovascular risk factors
1	63	M	32.3	TX N2 M1a	< 1	1	Ex smoker	Hypertension, obesity
2	59	M	20.8	T4 N3 M0	70	1	Ex smoker	–
3	53	F	27.4	T4 N3 M1c	55	0	Ex smoker	–
4	66	F	23.4	T1b N0 M1b	< 1	0	Ex smoker	–
5	58	F	23.4	T3 NX M1b	90	1	Ex smoker	–
6	64	M	29.8	T4 N3 M1a	80	1	Ex smoker	Peripheral vascular disease
7	73	M	33.3	T4 N2 M1	55	1	Ex smoker	Chronic kidney disease, obesity
8	72	M	24.9	T1b N2 M1b	95	1	Ex smoker	Hypertension
9	75	F	32.6	T2a N2 M1b	< 1	1	Ex smoker	Cerebrovascular disease, obesity
10	59	M	24.2	T2a N2 M1a	< 1	0	Smoker	Diabetes mellitus

PD-L1 expression measured using SP263 assay

**Table 2:** Detailed breakdown of immunotherapy, and SUV measurements of the left and right ventricle and tumour

Patient	Immunotherapy	Chemotherapy	Baseline LV_max_:BP	Baseline RV_max_:BP	Heterogeneity index (LV_max_/LV_mean_)	Heterogeneity index (RV_max_/RV_mean_)	LV: tumour uptake LVmax:Tumourmax	RV: tumour uptake RV_max:_Tumour_max_
1	Pembrolizumab	Carboplatin and pemetrexed	2.55	1.64	1.54	1.53	0.86	0.55
2	Pembrolizumab	–	2.05	1.56	1.44	1.59	0.58	0.44
3	Pembrolizumab	–	2.46	1.68	1.92	1.81	1.07	0.73
4	Pembrolizumab	Carboplatin and pemetrexed	2.96	1.69	1.58	1.60	0.43	0.25
5	Pembrolizumab	Carboplatin and pemetrexed	3.67	2.19	1.93	1.58	1.08	0.64
6	Pembrolizumab	–	1.67	1.25	1.67	1.66	0.37	0.28
7	Pembrolizumab	–	2.36	2.17	1.51	1.94	0.40	0.36
8	Pembrolizumab	–	3.08	1.88	1.43	1.44	0.76	0.47
9	Pembrolizumab	Carboplatin and pemetrexed	2.96	1.87	1.71	1.81	0.84	0.53
10	Pembrolizumab	Carboplatin and pemetrexed	3.80	2.27	1.71	1.62	1.39	0.83

BP: Blood pool ROI. LV_max_: Left ventricular maximum ROI. RV: Right ventricular maximum

**Fig. 2:**
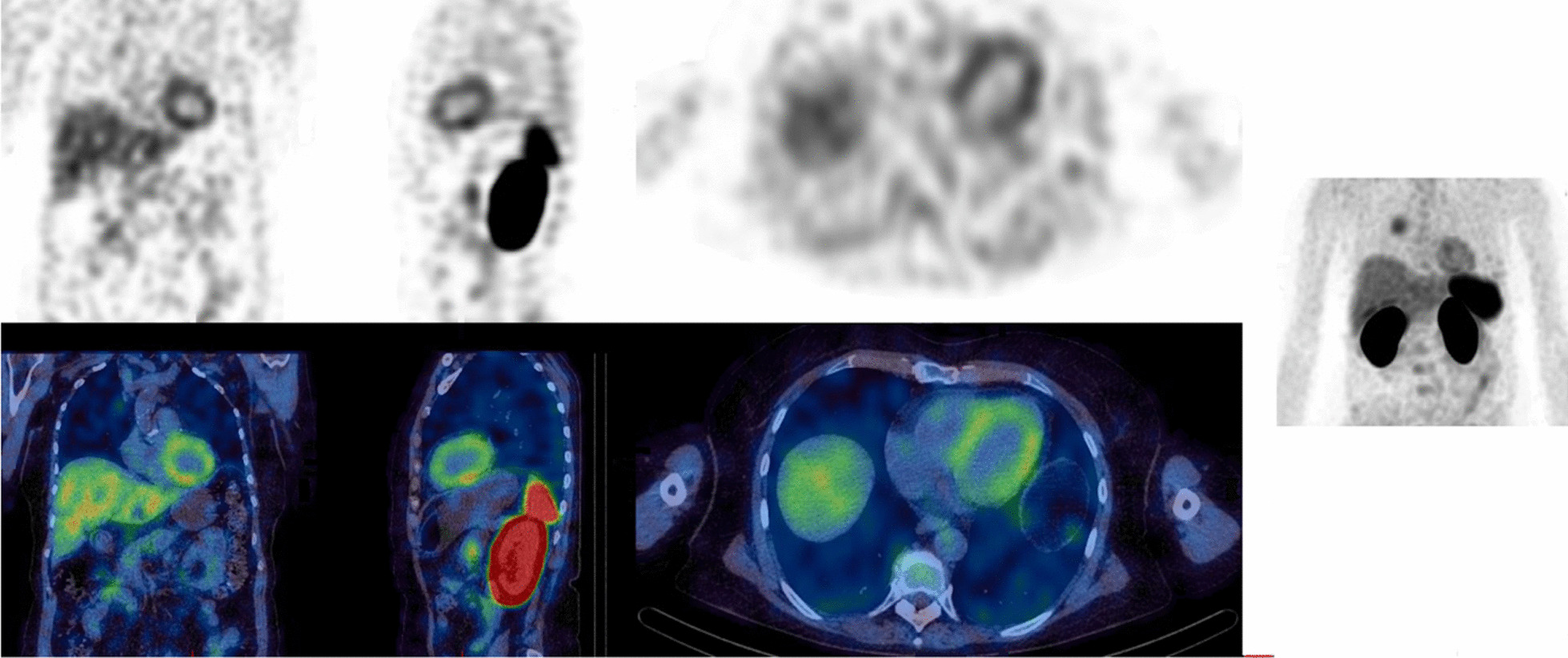
Baseline [^99m^Tc]NM-01 SPECT/CT images for a patient with non-small cell lung cancer. In addition to the uptake of [^99m^Tc]-labelled anti-PD-L1 single-domain antibody (NM-01) in the right lung tumour, tracer uptake is seen in the myocardium. The left ventricular: blood pool maximum region of interest (ROI_max_) ratio was 3.1, and the right ventricular: blood pool ratio was 1.5. In addition, there is a normal distribution of hepatic, bone marrow, splenic and renal activity. Very low muscle activity is present in the paraspinal muscles, in contrast to the increased myocardial PD-L1 expression

For baseline and 9-week follow-up scans, myocardial PD-L1 expression was evident in all patients. A typical case example is shown in Fig. 2. The mean administered activity at baseline and at 9 weeks was 612 ± 71 MBq and 660 ± 100 MBq, respectively, which was not significantly different (*p* = 0.32).

The mean LV_max_:BP was 2.76 ± 0.67 at baseline and 2.55 ± 0.77 at 9 weeks, which was not statistically different (*p* = 0.42). Mean RV_max_:BP was 1.82 ± 0.32 at baseline and 1.76 ± 0.45 at 9 weeks, the difference was not statistically significant (*p* = 0.67).

There was inter-individual heterogeneity for PD-L1 expression as determined by the HI. The median (range) for LV_max_:LV_mean_ HI values was 1.63 (1.43–1.93) at baseline and 1.58 (1.39 – 2.03) at 9 weeks. However, the differences between baseline and the 9-week scans were not statistically significant (*p* = 0.91). Median (range) RV_max_:RV_mean_ HI values were 1.61 (1.44 – 1.94) at baseline and 1.62 (1.35 – 2.49) at 9 weeks, which were not statistically different (*p* = 0.75). There was no difference in the HI between the left and right ventricle at baseline (*p* = 0.85) or follow-up (*p* = 0.75).

### Specificity of tracer to PD-L1 activity

At baseline, the mean LV_max_ was statistically greater than the mean muscle_max_ (3.71 ± 0.77 vs 0.98 ± 0.20 (*p* < 0.001)). At 9-week follow-up, the mean LV_max_ was significantly greater than the mean muscle_max_ (3.62 ± 1.38 vs 0.87 ± 0.19 (*p* < 0.001)). Overall, the mean LV_max_: muscle_max_ was 3.84 ± 0.73 and 4.09 ± 1.00, at baseline and 9-week follow-up, respectively, and this difference was not statistically significant (*p* = 0.29).

At baseline, the mean RV_max_ was significantly greater than the mean muscle_max_ (2.49 ± 0.63 vs 0.98 ± 0.20 (*p* < 0.001)). At 9-week follow-up, the mean RV_max_ was significantly greater than the mean muscle_max_ (2.50 ± 0.82 vs 0.87 ± 0.19 (*p* < 0.001)). Overall, the mean RV_max_: muscle_max_ was 2.59 ± 0.62 and 2.85 ± 0.61, at baseline and 9-week follow-up, respectively, and this difference was not statistically significant (*p* = 0.48).

### Measurement reliability

**Fig. 3:**
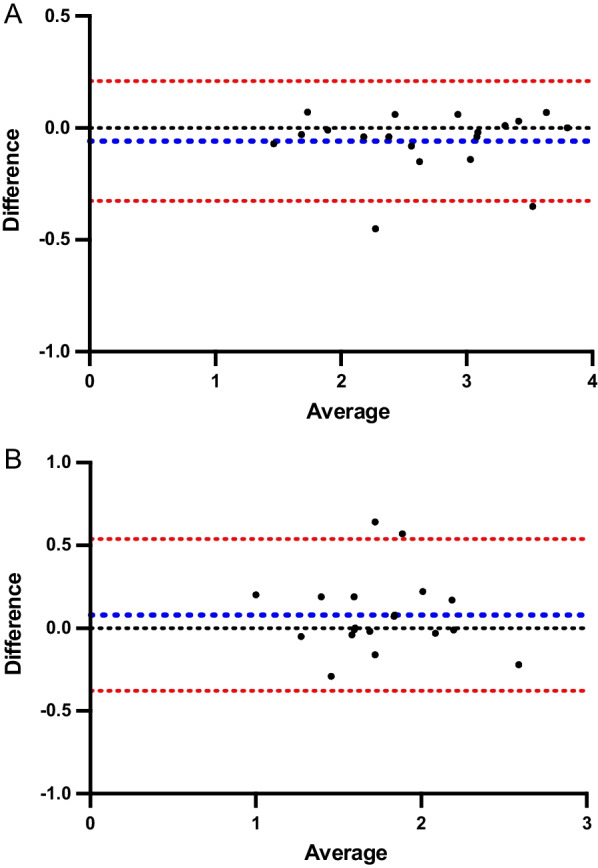
Bland-and-Altman plots that demonstrate intra-rater reliability for ROI_max_ ratios for [^99m^Tc]-labelled anti-PD-L1 single-domain antibody (NM-01) SPECT/CT (**A**) left ventricular: blood pool and (**B**) right ventricular: blood pool. (Blue dotted line: mean bias, red dotted lines: 95% limits of agreement)

For LV_max_:BP, there was excellent intra-rater reliability with ICC 0.99 (95% confidence interval 0.94–0.99, *p* < 0.001). The mean bias of measurements was -0.05 ± 0.14 with 95% limits of agreement -0.32 to 0.21, as shown in the Bland-and-Altman plot (Fig. 3A).

For RV_max_:BP, there was good intra-rater reliability with ICC 0.81 (95% confidence interval 0.57–0.92, *p* < 0.001). The mean bias of measurements was 0.08 ± 0.23 with 95% limits of agreement -0.38 to 0.54, as shown in the Bland-and-Altman plot (Fig. 3B).

### Clinical follow-up and adverse cardiovascular events

During this follow-up period, there was no evidence of MACE or suspected ICI-related myocarditis in this cohort of patients. One patient developed a large pericardial effusion, which required semi-urgent percutaneous pericardial drainage. For this patient, the serum troponin was not elevated and had rapidly progressive disease of the underlying cancer, and thus, the clinical presentation was more likely related to a malignant pericardial effusion rather than due to an inflammatory aetiology.

## Discussion

This is the first reported clinical study, to the authors’ knowledge, which confirms myocardial PD-L1 expression in the human heart using non-invasive imaging in vivo. We used a novel SPECT tracer, [^99m^Tc]NM-01, which is highly sensitive and specific for PD-L1, for the assessment of PD-L1 expression in patients with NSCLC [8, 9]. We demonstrated the ability to non-invasively quantify PD-L1 activity in the myocardium, that heterogeneity of expression exists within and between individuals and that there was no significant difference in measured PD-L1 expression on serial imaging during ICI therapy in this cohort. Moreover, we confirmed PD-L1 expression of the myocardium was greater than skeletal muscle, thus lending further support for the specificity of this tracer for PD-L1 expression.

Previous studies have reported that positron emission tomography (PET) tracers such as [^18^F]BMS-986192 and [^89^Zr]atezolizumab can be used to non-invasively quantify PD-L1 expression in NSCLC tumours in humans [16, 17]. However, these studies have not reported on cardiac PD-L1 expression. Mouse models have shown that PD-L1 expression has an important role in cardiac disease. One study reported that mice with a genetic deletion of PD-1 ligands that were treated with PD-L1 antibody therapy resulted in death from induced myocarditis [18]. Additionally, PD-1 expression has shown to have a protective effect as PD-1 deficient mice had increased myocardial inflammation and inflammatory cell infiltration in a model of experimental myocarditis [19]. PD-1 knockout mice developed severely impaired biventricular systolic function as measured with echocardiography and were found to have immunoglobulin deposition on the cardiomyocytes [20]. This later study suggested that PD-1 may have a protective role in the development of dilated cardiomyopathy from autoimmune disease. More recently, PD-L1 expression has been implicated in acute cellular rejection following heart transplantation [21]. Thus, PD-L1 expression is likely to have an important role in a range of cardiac disease, but these studies have been confined to preclinical experimental animal studies or those requiring invasive myocardial biopsy. Our study is the first to confirm the presence and expression of PD-L1 in myocardium non-invasively in the human heart in vivo. [^99m^Tc]NM-01 has favourable imaging properties as it binds to a different domain of PD-L1 to therapeutic monoclonal antibodies, which means it should not be blocked by anti-PD-L1 immunotherapy agents [9]. Our study provides an opportunity to apply the novel imaging biomarker [^99m^Tc]NM-01 SPECT/CT to assess the biological activity of PD-L1 non-invasively, which may be applied to obtain mechanistic understanding of a wide range of cardiac diseases.

Immune checkpoint inhibitors such as pembrolizumab block the PD-1 pathway; a potential mechanism for subsequent myocarditis may relate to blocking these protective pathways on the cardiomyocytes. [^99m^Tc]NM-01 may be used to understand the mechanism of ICI-associated myocarditis, which is associated with T cell-mediated infiltration of the myocardium [22]. ICI-associated myocarditis is a challenging clinical diagnosis and requires the integration of symptoms, blood biomarkers such as troponin and cardiovascular magnetic resonance (CMR) or ^18^F-FDG PET imaging [23]. Endomyocardial biopsy is recommended in cases when there are uncertain CMR or PET findings or the patient cannot undergo non-invasive assessment due to hemodynamic instability [24]. However, sampling error is a well-known limitation of endomyocardial biopsy due to the heterogeneous myocardial pattern of various cardiac diseases, and therefore, it is recommended that at least five samples should be taken from different sites [25]. The issue of heterogeneity may potentially be overcome with non-invasive imaging, which provides an assessment of complete myocardial coverage and thus may allow global myocardial assessment of PD-L1 expression and heterogeneity compared to endomyocardial biopsy.

Importantly, there is no validated risk prediction tool, or biomarker that can accurately predict which patients are at highest risk of developing ICI-associated myocarditis. It may be plausible that the non-invasive quantification of PD-L1 expression in the myocardium may be used as an imaging biomarker to predict which patients are at highest risk of myocarditis. For instance, based on the importance of PD-1 in mouse models, it may be possible that patients with low PD-L1 expression at the time of cancer diagnosis and before ICI treatment are at increased risk of ICI-related myocarditis. However, future clinical studies are required to understand this association. This has potential clinical application as it may aid in the identification of patients at highest risk of developing myocarditis in the future, and guide diagnosis and frequency of surveillance for cardiovascular toxicity in patients undergoing immunotherapy.

Another potential application for the assessment of PD-L1 myocardium expression is in patients with dilated cardiomyopathy. There are various causes of dilated cardiomyopathy, which include genetic, toxins, idiopathic, infective, inflammatory, infiltrative and autoimmune conditions [26]. In the extensive workup of such patients, often no clear underlying aetiology is demonstrated and thus considered to be idiopathic. Knockout PD-1 mice have been shown to develop biventricular systolic dysfunction [20]. One potential avenue to consider is whether patients with dilated cardiomyopathy may have reduced PD-L1 activity, which may be investigated in a well-characterized cohort of patients. This may aid understanding of the pathophysiological mechanism in dilated cardiomyopathy and thus guide protective medical therapy to prevent the development of adverse left ventricular remodelling.

There are some important limitations to acknowledge from this study. Firstly, this was a single-centre study with a small number of patients, but nevertheless consistently demonstrates the first in man feasibility of this imaging biomarker to measure myocardial PD-L1 expression. Secondly, patients with established NSCLC were considered, rather than a wider range of cancers. Thirdly, there was no control group of healthy volunteers to determine the potential effect of active malignancy on myocardial PD-L1 expression. Finally, an invasive biopsy of the myocardium was not obtained, which would have been useful to confirm human myocardial PD-L1 expression at the gene and protein levels and the cells where expression is observed. However, previous preclinical data confirmed high specificity of [^99m^Tc]NM-01 for PD-L1 [8], our previous study demonstrated good correlation with immunohistochemistry PD-L1 in lung cancer patients [9] and this current study showed high myocardial activity compared to striated muscle, which all provide strong indirect evidence of PD-L1 specificity in the myocardium for [^99m^Tc]NM-01. The physical limitations of SPECT compared to PET with regard to spatial resolution are acknowledged, but nevertheless our image data confirmed good myocardial to background contrast and was therefore readily quantifiable and reproducible. Potential further improvements could be made with cardiac and respiratory gating.

## Conclusion

Myocardial PD-L1 expression can be identified non-invasively with [^99m^Tc]NM01 SPECT/CT. This quantitative imaging biomarker has excellent reliability, and a threefold-to-fourfold magnitude in measurement compared to striated muscle tissue, in keeping with the known high specificity to PD-L1 expression. This study thus paves the way to undertake clinical studies to ascertain the biological role and clinical significance of PD-L1 expression in the heart in a wide range of cardiac disease, such as ICI-associated myocarditis and cardiomyopathies.

## Data Availability

The datasets used and/or analysed during the current study are available from the corresponding author on reasonable request.
